# Influence of Anterior Cruciate Ligament Tear on Thigh Muscle Strength and Hamstring-to-Quadriceps Ratio: A Meta-Analysis

**DOI:** 10.1371/journal.pone.0146234

**Published:** 2016-01-08

**Authors:** Hyun-Jung Kim, Jin-Hyuck Lee, Sung-Eun Ahn, Min-Ji Park, Dae-Hee Lee

**Affiliations:** 1 Department of Preventive Medicine, Korea University College of Medicine, Seoul, Korea; 2 Department of Sports Medical Center, Korea University College of Medicine, Seoul, Korea; 3 Department of Orthopaedic Surgery, Samsung Medical Center, Sungkyunkwan University School of Medicine, Seoul, Korea; Rutgers University -New Jersey Medical School, UNITED STATES

## Abstract

Theoretical compensation after anterior cruciate ligament (ACL) tear could cause quadriceps weakness and hamstring activation, preventing anterior tibial subluxation and affecting the expected hamstring-to-quadriceps ratio. Although quadriceps weakness often occurs after ACL tears, it remains unclear whether hamstring strength and hamstring-to-quadriceps ratio increase in ACL deficient knees. This meta-analysis compared the isokinetic muscle strength of quadriceps and hamstring muscles, and the hamstring-to-quadriceps ratio, of the injured and injured limbs of patients with ACL tears. This meta-analysis included all studies comparing isokinetic thigh muscle strengths and hamstring-to-quadriceps ratio in the injured and uninjured legs of patients with ACL tear, without or before surgery. Thirteen studies were included in the meta-analysis. Quadriceps and hamstring strengths were 22.3 N∙m (95% CI: 15.2 to 29.3 N∙m; P<0.001) and 7.4 N∙m (95% CI: 4.3 to 10.5 N∙m; P<0.001) lower, respectively, on the injured than on the uninjured side. The mean hamstring-to-quadriceps ratio was 4% greater in ACL deficient than in uninjured limbs (95% CI: 1.7% to 6.3%; P<0.001). Conclusively, Decreases were observed in both the quadriceps and hamstring muscles of patients with ACL tear, with the decrease in quadriceps strength being 3-fold greater. These uneven reductions slightly increase the hamstring-to-quadriceps ratio in ACL deficient knees.

## Introduction

Following injury to the anterior cruciate ligament (ACL), isokinetic quadriceps strength is generally lower in the injured than in the contralateral uninjured knee due to arthrogenic muscle inhibition.[1–3] This phenomenon is considered a natural compensating mechanism to prevent anterior subluxation that may result in painful and potentially detrimental movements of the injured knee.[4] This compensating mechanism may include the facilitation or activation of the knee flexor (hamstring),[5]in addition to quadriceps inhibition. The activated hamstring could thereby counteract anterior shear forces during knee loading.[6,7].

However, it is unclear whether the isokinetic muscle strength of the hamstring is also reduced after ACL tear, and, if so, whether the magnitude of reduction is similar to that of the quadriceps muscle. Furthermore, the balance between the quadriceps and hamstring muscles, usually assessed as the hamstring-to-quadriceps (HQ) ratio, may be altered in knees with ACL tears when compared with contralateral uninjured knees. Such alterations in the HQ ratio,[8]indicating an imbalance between the quadriceps and hamstring strengths, may increase the risk of further injury of the lower limb and osteoarthritis after ACL injury. [9,10] Adequate restoration of hamstring and quadriceps strength after ACL injury or reconstruction could be used as a parameter for decision-making regarding return to sports. [11] Therefore, an accurate understanding of HQ ratio alteration in patients with ACL tears is important. However, previous studies[12,13] investigating the HQ ratio in patients with ACL tears have yielded conflicting results.

Therefore, this meta-analysis compared the isokinetic muscle strengths of the quadriceps and hamstring muscles, as well as the HQ ratio, of injured limbs with ACL tears and contralateral uninjured limbs. This study hypothesized that, although the isokinetic strengths of both muscles would be lower on the injured than on the uninjured side, the HQ ratio would be relatively unchanged because the magnitude of decrease of both muscles on the injured side would be similar.

## Materials and Methods

This meta-analysis was performed according to the guidelines of the preferred reporting items for systematic reviews and meta-analysis (PRISMA) statement (S1 PRISMA Checklist).

### Data & Literature Sources

This study was based on Cochrane Review Methods.[14] Multiple comprehensive databases, including MEDLINE (January 1, 1976 to April 30, 2014), EMBASE (January 1, 1985 to June 30, 2014), the Cochrane Library (January 1, 1987 to June 30, 2014) and KoreaMed (June 1, 1958 to June 30, 2014), were searched for studies that compared isokinetic muscle strengths of the quadriceps and/or hamstring muscles, and/or the HQ ratio, between limbs with ACL tears and contralateral uninjured limbs. There were no restrictions on language or year of publication. Search terms used in the title, abstract, MeSH and keywords fields included "Anterior Cruciate Ligament" [tiab] OR "Anterior Cruciate Ligaments" [tiab] OR "ACL" [tiab], and "Muscle Strength" [MeSH] OR "Muscle Contraction" OR "Isometric Contraction" [MeSH] OR "Quadriceps" [tiab] OR "Muscle Contractions" [tiab] OR "Muscular Contraction" [tiab] OR "Muscular Contractions" [tiab] OR "Isometric Contractions" [tiab] OR "hamstring" [tiab] OR "hamstrings" [tiab]. After the initial electronic search, we hand-searched additional relevant articles and the bibliographies cited by identified studies. Articles identified were assessed individually for inclusion.

### Study Selection

Study inclusion was decided independently by two reviewers, based on the predefined selection criteria. Titles and abstracts were read; if suitability could not be determined, the full article was evaluated. Studies were included in the meta-analysis if (1) they analyzed patients with ACL tears, without or before surgery, (2) they reported direct comparisons of isokinetic thigh muscle concentric strength, including quadriceps and hamstring muscles, and/or their concentric (HQ) ratio of injured limbs with ACL tear and contralateral uninjured limbs, (4) they measured isokinetic thigh muscle strength with a dynamometer as maximal peak torque, (5) they fully reported the means and standard deviations of the isokinetic strength of thigh muscles and their HQ ratio, and sample numbers, and (5) they used adequate statistical methods to compare muscle strengths and HQ ratios of injured and uninjured limbs.

Studies that measured muscle strength using isometric tests were excluded, because the results of such tests may be affected by the learning curve effect and have lower reliability compared with isokinetic tests.

### Data Extraction

Two reviewers independently recorded data from each study using a predefined data extraction form. Any disagreement unresolved by discussion was reviewed by a third author.

Variables recorded included: (1) means and standard deviations of isokinetic muscle strength (maximal peak torque) of the quadriceps and hamstrings, and HQ ratio, of injured and uninjured limbs; (2) sample size; and (3) angular velocity, a measure of maximal peak torque. If these variables were not mentioned in the articles, we contacted the study authors by email to request the data.

### Assessment of Methodological Quality

Two reviewers independently assessed the methodological quality of each study using the Newcastle-Ottawa Scale,[15] as recommended by the Cochrane Non-Randomized Studies Methods Working Group. For our purposes, we adjusted the Newcastle-Ottawa Scale’s star system, which awards stars depending on level of bias, to a scale that included only low (one star), high, and unclear bias. Each study was judged on three criteria: the selection of the study groups, the comparability of the groups and the ascertainment of either the exposure or the outcome of interest for case-control or cohort studies. Any unresolved disagreements between reviewers were resolved by consensus or by consultation with a third investigator.

### Statistical Analysis

The main outcome of the meta-analysis was the mean difference in isokinetic concentric strength (maximal peak torque) of the quadriceps and hamstring muscles, and the HQ ratio, of injured and uninjured limbs. Random-effects meta-analyses were used to pool these outcomes across the included studies, estimating weighted mean differences in thigh muscle strength and their ratio between injured and uninjured limbs and their associated 95% confidence intervals (CIs). Heterogeneity was determined by estimating the proportion of between-study inconsistencies due to actual differences between studies, rather than differences due to random error or chance, using the I^2^ statistic, with values of 25%, 50%, and 75% considered low, moderate, and high, respectively. To examine whether the demographic characteristics of participants had a biasing effect on any of the effect sizes, including the differences in hamstring and quadriceps muscle strengths and their ratio between uninvolved and involved legs, meta-regression analyses were conducted with effect size as the dependent variable and age and gender ratio as predictors. All statistical analyses were performed using RevMan version 5.2 and Stata/MP 13.0. A *P* value < .05 was considered significant.

## Results

### Identification of Studies

**Fig 1:**
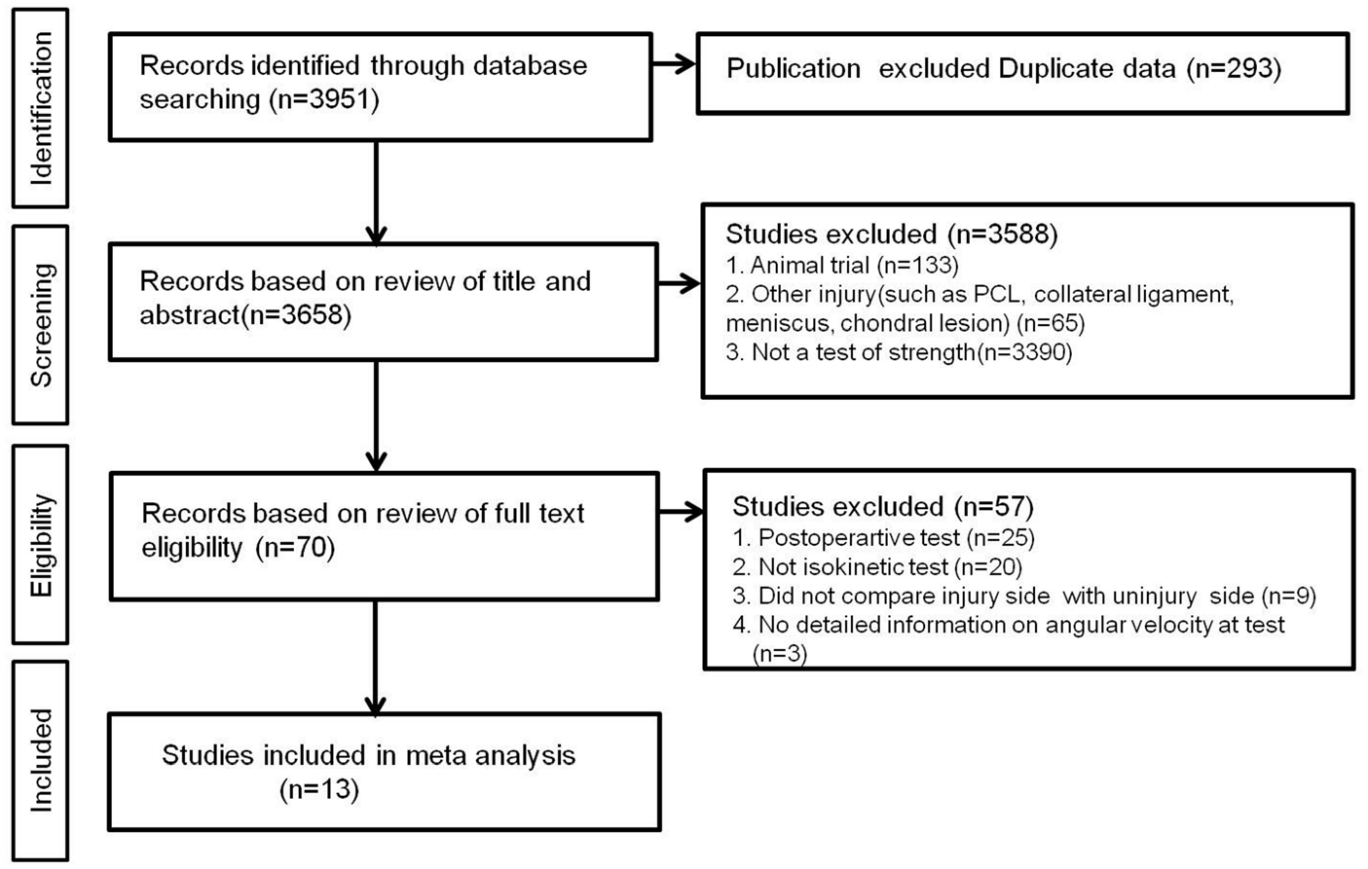
PRISMA (Preferred Reporting Items for Systematic reviews and Meta-analyses) flow diagram of the identification and selection of the studies included in this meta-analysis.

Fig 1 shows the details of study identification, inclusion, and exclusion. An electronic search yielded 423 studies in PubMed (MEDLINE), 426 in EMBASE, 48 in the Cochrane Library and 20 in KoreaMed. Five additional publications were identified through manual searching. After removing 339 duplicates, 583 studies remained; of these, 556 were excluded based on reading of the abstracts and full-text articles and an additional 13 studies were excluded based on unusable information and inappropriate group comparisons. Only the most recently published of two papers describing the same study was included in the analysis. After applying these criteria, 13 studies were included in this meta-analysis.

### Study Characteristics and Patient Populations

The 13 included studies included 18 patient cohorts and 548 knees with ACL tears, with thigh muscle strength or HQ ratio measured for each knee. Of the 13 studies, eight prospectively compared results in the injured and noninjured sides, whereas the other five were retrospective comparisons. Five studies evaluated both thigh muscle strength and HQ ratio; six compared only thigh muscle strength; and two compared only HQ. Ten of the 12 studies measured thigh muscle strength and HQ ratio at 60°/sec, as well as angular velocity at 120°/sec, 180°/sec, and/or 270°/sec. Of the remaining three studies, two out of them measured HQ ratio at an angular velocity of 30°/sec and 60°/sec, respectively, and one study measured thigh muscle strengths at angular velocities of 30°/sec and 90°/sec. The mean time interval from injury to measurement varied from two to 33 months, indicating that most studies included patients with chronic ACL tears (Table 1).

**Table 1:** Study characteristics.

Authors	Year	Study type	Sample size	Measured Parameters (angular velocity)	Mean time interval from injury to testing (months)
Benjuya et al.[20]	2000	PCS	27	Q(60°/sec, 180°/sec), H(60°/sec, 180°/sec), HQ ratio (60°/sec, 180°/sec)	9
Dvir et al.[21]	1989	RCS	35	HQ ratio(30°/sec)	11
Eitzen et al.[22]	2013	PCS	100	Q(60°/sec), H(60°/sec)	2
Friden et al.[23]	2010	RCS	18	Q (30°/sec, 90°/sec), H(30°/sec, 90°/sec)	29
Gibson et al.[13]	2000	RCS	18	Q(60°/sec), H(60°/sec)	NC
Hole et al.[12]	2000	PCS	10	HQ ratio (60°/sec)	NC
Kannus et al.[24]	1988	RCS	41	Q(60°/sec, 180°/sec), H(60°/sec, 180°/sec), HQ ratio (60°/sec, 180°/sec)	NC
Keays et al.[25]	2003	RCS	31	Q (60°/sec, 120°/sec), H(60°/sec, 120°/sec)	33
Lee HM et al.[26]	2009	PCS	12	Q(60°/sec), H(60°/sec), HQ ratio (60°/sec)	17.5
Lee JC et al.[27]	2013	PCS	10	Q(60°/sec), H(60°/sec)	NC
Lephart et al.[28]	1992	PCS	41	Q(60°/sec, 270°/sec), H(60°/sec, 270°/sec), HQ ratio (60°/sec, 270°/sec)	26.5
Segawa et al.[29]	2002	PCS	62	Q(60°/sec), H(60°/sec)	10.2
Tsepis et al.[30]	2004	PCS	30	Q(60°/sec), H(60°/sec), HQ ratio (60°/sec)	32

Abbreviations: PCS, prospective comparison study; RCS, retrospective comparison study; Q, quadriceps; H, hamstring; HQ, hamstring-to-quadriceps.

### Quality and Publication bias of the Included Studies

All 13 studies included in this meta-analysis were at low risk of selection bias. All compared injured legs with ACL tears with contralateral uninjured legs as controls, and 11 provided detailed demographic data. None assessed possible confounding factors. The evaluation of follow up duration was modified relative to the chronic nature of ACL (time from injury to measurement). We assumed that too short an interval from injury (<4 weeks) was insufficient to measure isokinetic muscle strength due to the pain usually caused by limited motion of the knee joint. Nine studies evaluated patients >2 months after injury, indicating that all included patients could perform isokinetic tests appropriately without pain. However, the remaining studies, which did not report the chronicity of ACL tear, were regarded as biased. None of the included studies mentioned the percentage of patients evaluated, relative to all patients with ACL tears who visited each institution. We therefore deemed all studies included in this meta-analysis as having a high risk of bias (Table 2).

**Table 2:** Risk of bias summary: authors’ judgments about each risk of bias item for each included study.

Author	Representativeness of the cases	Selection of controls	Ascertainment of exposure	Interest outcome not present at start of study	Comparability of cohorts	Control for any additional factor	Assessment of outcome	Enough Follow- up	Adequacy of follow up
Benjuya et al.[20]	−	−	−	−	−	+	+	−	+
Dvir et al.[21]	−	−	−	−	+	+	+	−	+
Eitzen et al.[22]	−	−	−	−	−	+	+	−	+
Friden et al.[23]	−	−	−	−	−	+	+	−	+
Gibson et al.[13]	−	−	−	−	−	+	+	+	+
Hole et al.[12]	−	−	−	−	−	+	−	+	+
Kannus et al.[24]	−	−	−	−	−	+	+	+	+
Keays et al.[25]	−	−	−	−	−	+	+	−	+
Lee HM et al.[26]	−	−	−	−	−	+	−	−	+
Lee JC et al.[27]	−	−	−	−	−	+	+	+	+
Lephart et al.[28]	−	−	−	−	−	+	_+_	−	+
Segawa et al.[29]	−	−	−	−	−	+	+	−	+
Tsepis et al.[30]	−	−	−	−	−	+	−	−	+

−, low risk of bias; +, high risk of bias;?, unclear risk of bias

### Isokinetic Quadriceps Strength

**Fig 2:**
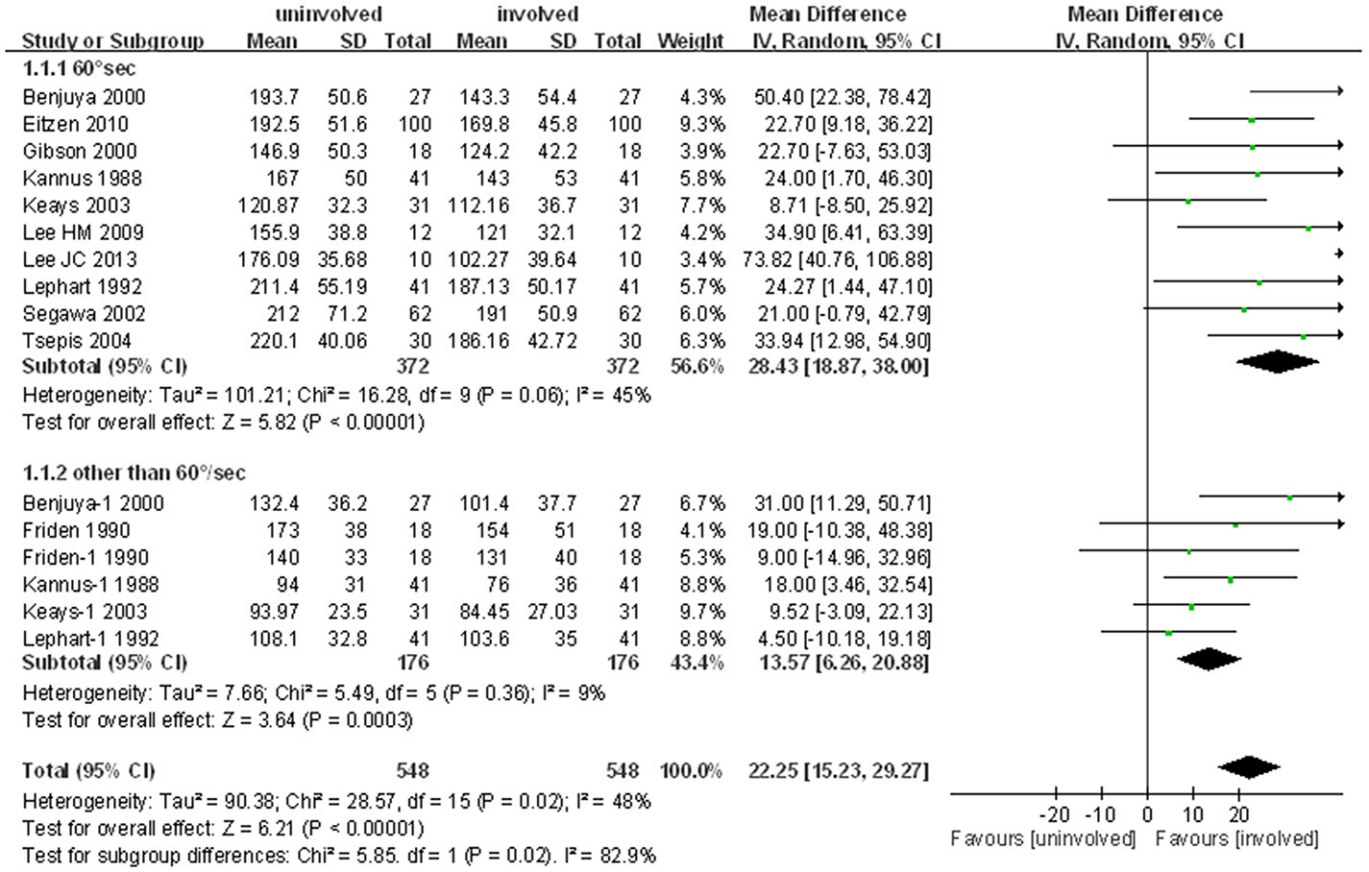
Forest plot demonstrating significant reductions in quadriceps strength in ACL deficient limbs compared with uninjured limb.

Eleven studies, including 16 comparison cohorts, compared the isokinetic strength of the quadriceps muscles of injured and uninjured legs. The pooled standard mean difference in mean peak torque of the quadriceps muscles of the two legs was 22.3 N∙m (95% CI: 15.2 to 29.3 N∙m; P<0.001; I^2^ = 48%), indicating that the isokinetic quadriceps strength was lower on the injured side than on the uninjured side. Subgroup analysis of the ten studies assessing quadriceps strength at 60°/sec showed that quadriceps strength was 28.4 N∙m lower on the injured than on the uninjured side (95% CI: 18.9 to 38.0 N∙m; P<0.001; I^2^ = 45%). Subgroup analysis, involving six cohorts in five studies, including two cohorts at 180°/sec and one each at 30°/sec, 90°/sec, 120°/sec and 270°/sec, showed similar results, with mean quadriceps strength being 13.6 N∙m lower on the injured than on the uninjured side (95% CI: 6.3 to 20.9; P<0.001; I^2^ = 9%, Fig 2).

### Isokinetic Hamstring Strength

Similar results were observed in the meta-analysis of isokinetic hamstring strength, which involved 11 studies and 16 cohorts, comparing the isokinetic strength of the hamstring muscles of the injured and uninjured legs.

**Fig 3:**
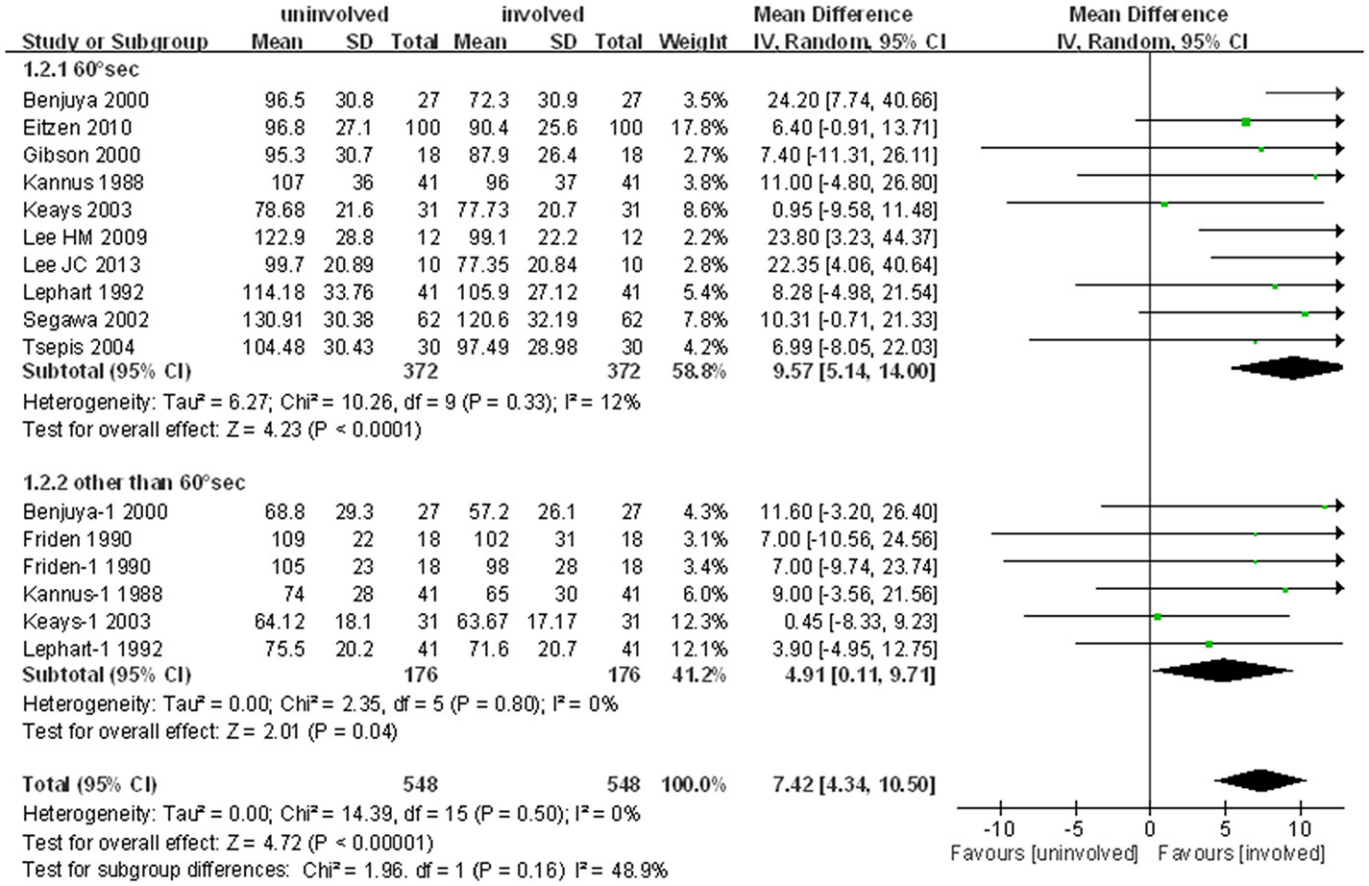
Forest plot showing significant reductions in hamstring strength in ACL deficient compared with uninjured limbs.

The pooled standard mean difference in mean peak torque of the hamstring muscles of the two legs was 7.4 N∙m (95% CI: 4.3 to 10.5 N∙m; P<0.001; I^2^ = 0%). Subgroup analysis of the ten studies in which strength was measured at 60°/sec also showed a significant mean difference (9.6 N∙m, 95% CI: 5.1 to 14.0 N∙m; P<0.001; I^2^ = 12%) between the injured and uninjured limbs. Similarly, subgroup analysis of the six other cohorts in five studies, two cohorts at 180°/sec and one each at 30°/sec, 90°/sec, 120°/sec and 270°/sec, showed that isokinetic hamstring strength was 4.9 N∙m lower in the injured than the uninjured limb (95% CI: 0.1 to 9.7 N∙m; P = 0.04; I^2^ = 0%, Fig 3). These findings demonstrated that, like quadriceps strength, hamstring strength was lower in legs with than without ACL tears. However, the reduction in hamstring strength was only one-third the reduction in quadriceps strength.

### Hamstring-to-Quadriceps Ratio

**Fig 4:**
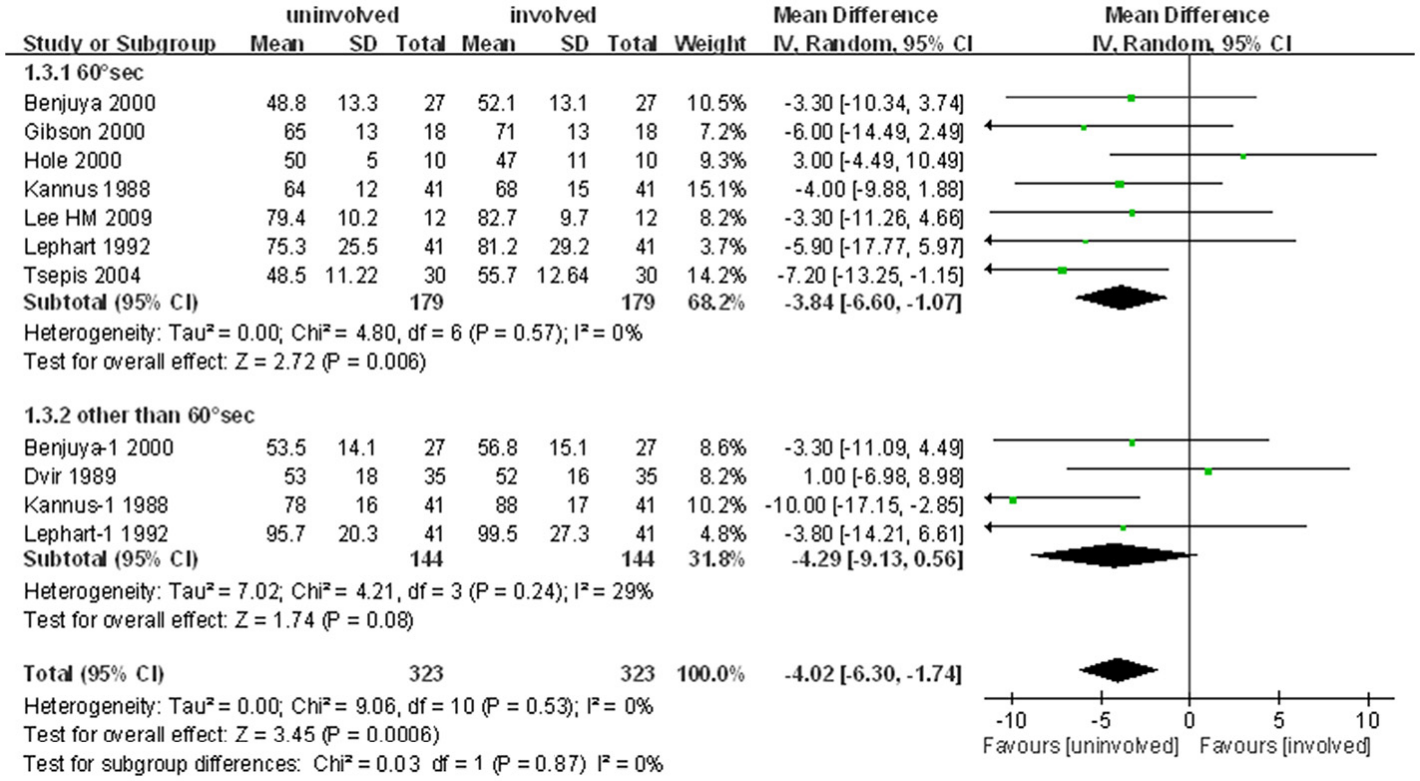
Forest plot demonstrating slight reductions in hamstring-to-quadriceps ratio in ACL deficient compared with uninjured limbs.

Eight studies, including eleven comparison cohorts, compared the HQ ratio of injured and uninjured legs. The pooled standard mean difference in mean peak torque of the HQ ratio was 4.0% (95% CI: 1.7% to 6.3%; P<0.001; I^2^ = 0%), indicating that the HQ ratio was slightly higher in the injured than the uninjured leg. Analysis of the 7 studies in which HQ ratio was measured at 60°/sec also showed a slight increase (3.8%, 95% CI: 1.1% to 6.6%; P = 0.006; I^2^ = 0%) on the injured side. Analysis of the 4 cohorts measured at other than 60°/sec (two cohorts at 180°/sec, one at 30°/sec and one at 270°/sec) found that the HQ ratio was 4.3% (95% CI: 0.6% to 9.1%; I^2^ = 29%) higher on the injured than on the uninjured side. This difference approached statistical significance but was not statistically significant (P = 0.08, Fig 4).

### Metaregression analyses

The results of the meta-regression analysis are reported in Table 3. No significant association was found between age and gender, and any of the effect sizes, including the differences in hamstring and quadriceps muscle strengths and their ratio between uninvolved and involved legs. This finding indicated that the results of the current meta-analysis were not biased by the demographic characteristics of the participants of the included studies.

**Table 3:** Meta-regression analysis between the demographic variables age and gender, and thigh muscle strength parameters.

Variable	Coefficient	Standard error	P value	95% confidence interval
Difference of Hamstring muscle strength between uninvolved and involved leg				
Age	−0.068	1.288	0.959	−3.111 to 2.978
Gender	17.126	17.241	0.354	−23.643 to 57.895
Difference of Hamstring muscle strength between uninvolved and involved leg				
Age	−0.128	0.608	0.839	−1.568 to 1.311
Gender	4.546	8.056	0.590	−14.503 to 23.597
Difference of Hamstring-to-quadriceps ratio between uninvolved and involved leg				
Age	0.302	0.333	0.417	−0.624 to 1.229
Gender	−3.300	4.425	0.497	−15.588 to 8.986

## Discussion

This meta-analysis analyzed the reductions in quadriceps and hamstring muscle strengths, and their ratio, in ACL deficient knees. The main findings were that the reduction in quadriceps muscle strength was three-fold greater than the reduction in hamstring strength in knees with ACL tears, resulting in a slight increase in HQ ratio.

Quadriceps strength was shown to be altered in patients with ACL tears, a type of compensatory mechanism to reduce the anterior tibial subluxation caused by a torn ACL. Patients with ACL tears tend to avoid quadriceps contraction when the knee is near full extension, since this can induce strain on the ACL.[15] The reduction in quadriceps contraction observed in patients with ACL tears is likely to diminish the anterior translation of the tibia relative to the femur, protecting the knee from excessive anterior drawer. Therefore, quadriceps strength is generally reduced after ACL tear.[16,17] Although reduction in isokinetic strength of the quadriceps after ACL tear is regarded as a compensatory mechanism to avoid tibial anterior translation, the compensation mechanism also occurred in hamstring muscles. Increased hamstring strength is theoretically desirable in patients with ACL tears, because hamstring activation can reduce anterior tibial translation by decreasing the load on the passive restraints of the knee, increasing knee joint compression, and stabilizing the knee in response to an external varus/valgus load.[18,19] Thus, it was expected that the reductions in hamstring strength after ACL tears would not be as large as the reductions in quadriceps strength, but there is no consensus on the magnitude of hamstring reduction after ACL tear. This meta-analysis found that the reduction in quadriceps muscle strength was about 3-times greater than the reduction in hamstring muscle strength. These uneven reductions in thigh muscle strength contributed to muscle imbalance, as shown by the higher HQ ratio in injured than in uninjured limbs.

This meta-analysis also showed a greater loss in quadriceps than in hamstring dominant strength, resulting in thigh muscle imbalance, as shown by an increased HQ ratio in limbs of patients with ACL tear. This greater degree of loss of quadriceps strength may increase the knee adduction moment on the coronal plane during gait due to the greater reduction in abduction moment resulting from quadriceps weakness,[14] and may reduce the external knee flexion moment and knee flexion angle in the sagittal plane.[15,16] These changes in dynamic loading of the knee joint on both the coronal and sagittal planes could result in overloading of the knee joint due to the increased adduction moment on the coronal plane and the lack of shock absorption during weight-bearing, resulting from decreased flexion moment on the sagittal plane.[4] These situations may explain in part the development of knee osteoarthritis after chronic ACL tear or even in ACL reconstructed knees. Interestingly, however, the observed increase in HQ ratio in limbs of patients with ACL tears was significant only when measurements were performed at 60°/sec and not at other angular velocities. This dependence of the results on angular velocity may be explained in part by the fact that hamstring muscle strength measured at the higher angular velocities (180°/sec or 240°/sec) may have been slightly underestimated because the measurements were made very early (approximately 100 ms) after the onset of contraction, a time at which the contractile force likely had not reached its fully active state. This situation would lead to a smaller increase in HQ ratio at higher velocities compared with 60°/sec, consistent with the observation of a significantly greater HQ ratio only at 60°/sec.

This study had several limitations. The quality of the meta-analysis was dependent on the quality of the individual studies. Furthermore, all of the studies included in this meta-analysis were observational comparison studies; none of the studies was a randomized controlled trial. Thus, there may be inherent heterogeneity due to uncontrolled bias. In addition, the studies included in this meta-analysis compared thigh muscle strength in the limbs with and without ACL tears, rather than using a separate control group. Bilateral muscle strength impairment, utilizing aberrant information in the intra-articular receptors in the injured limb, may also decrease muscle strength in the contralateral, uninjured limb,[17] thus affecting the results of this meta-analysis. However, it is very difficult to select control subjects with the same activity level and athletic history as subjects with ACL tears. This meta-analysis also did not take into account possible gender differences in thigh muscle strength and HQ ratio in each included study. However, a recent study showed that gender differences in muscle strength and HQ ratio were not significant at slow testing speeds. We could not perform separate meta-analyses for males and females because none of the included studies provided separate data for males and females. Finally, it was unclear whether the slight difference (4%) in HQ ratio observed between ACL torn- and uninjured limbs has clinical implications. However, we believe that this small difference could affect the long term performance of ACL deficient knees, because even a small imbalance in hamstring to quadriceps strength could lead to lower extremity injury. In addition, the greater loss of quadriceps compared with hamstring strength observed in the current study demonstrates the importance of quadriceps strengthening exercises in ACL deficient knees.

## Conclusions

In conclusion, both the quadriceps and hamstring muscles lose strength in limbs with ACL tears. The loss of quadriceps strength was approximately three-fold greater than the loss of hamstring strength. The uneven reductions in the strengths of these thigh muscles resulted in a slight increase in HQ ratio in ACL deficient knees.

## Supporting Information

S1 PRISMA Checklist PRISMA checklist. (PDF)

## References

[1] Urbach D, Nebelung W, Becker R, Awiszus F. Effects of reconstruction of the anterior cruciate ligament on voluntary activation of quadriceps femoris a prospective twitch interpolation study. J Bone Joint Surg Br. 2001;83: 1104–1110. 11764420 10.1302/0301-620x.83b8.11618

[2] Palmieri RM, Weltman A, Edwards JE, Tom JA, Saliba EN, Mistry DJ, et al Pre-synaptic modulation of quadriceps arthrogenic muscle inhibition. Knee Surgery, Sports Traumatology, Arthroscopy. 2005;13: 370–376. 15685462 10.1007/s00167-004-0547-z

[3] de Jong SN, van Caspel DR, van Haeff MJ, Saris DB. Functional assessment and muscle strength before and after reconstruction of chronic anterior cruciate ligament lesions. Arthroscopy. 2007;23: 21–28, 28e21–23. 17210423 10.1016/j.arthro.2006.08.024

[4] Palmieri-Smith RM, Thomas AC. A neuromuscular mechanism of posttraumatic osteoarthritis associated with ACL injury. Exerc Sport Sci Rev. 2009;37: 147–153. 10.1097/JES.0b013e3181aa6669 19550206

[5] Georgoulis AD, Ristanis S, Moraiti CO, Paschos N, Zampeli F, Xergia S, et al ACL injury and reconstruction: Clinical related in vivo biomechanics. Orthop Traumatol Surg Res. 2010;96: S119–128. 10.1016/j.otsr.2010.09.004 21036116

[6] Ko MS, Yang SJ, Ha JK, Choi JY, Kim JG. Correlation between Hamstring Flexor Power Restoration and Functional Performance Test: 2-Year Follow-Up after ACL Reconstruction Using Hamstring Autograft. Knee Surg Relat Res. 2012;24: 113–119. 10.5792/ksrr.2012.24.2.113 22708113 PMC3373998

[7] Yoon JP, Yoo JH, Chang CB, Kim SJ, Choi JY, Yi JH, et al Prediction of chronicity of anterior cruciate ligament tear using MRI findings. Clin Orthop Surg. 2013;5: 19–25. 10.4055/cios.2013.5.1.19 23467216 PMC3582867

[8] Lee DH, Lee JH, Jeong HJ, Lee SJ. Lack of Correlation between Dynamic Balance and Hamstring-to-Quadriceps Ratio in Patients with Chronic Anterior Cruciate Ligament Tears. Knee Surg Relat Res. 2015;27: 101–107. 10.5792/ksrr.2015.27.2.101 26060609 PMC4458480

[9] Aagaard P, Simonsen EB, Magnusson SP, Larsson B, Dyhre-Poulsen P. A new concept for isokinetic hamstring: quadriceps muscle strength ratio. Am J Sports Med. 1998;26: 231–237. 9548116 10.1177/03635465980260021201

[10] Hortobagyi T, Westerkamp L, Beam S, Moody J, Garry J, Holbert D, et al Altered hamstring-quadriceps muscle balance in patients with knee osteoarthritis. Clin Biomech (Bristol, Avon). 2005;20: 97–104. 10.1016/j.clinbiomech.2004.08.004 15567543

[11] van Grinsven S, van Cingel RE, Holla CJ, van Loon CJ. Evidence-based rehabilitation following anterior cruciate ligament reconstruction. Knee Surg Sports Traumatol Arthrosc. 2010;18: 1128–1144. 10.1007/s00167-009-1027-2 20069277

[12] Hole CD, Smit GH, Hammond J, Kumar A, Saxton J, Cochrane T. Dynamic control and conventional strength ratios of the quadriceps and hamstrings in subjects with anterior cruciate ligament deficiency. Ergonomics. 2000;43: 1603–160. 11083140 10.1080/001401300750004023

[13] Clair Gibson A St, Lambert M, Durandt J, Scales N, Noakes T. Quadriceps and hamstrings peak torque ratio changes in persons with chronic anterior cruciate ligament deficiency. J Orthop Sports Phys Ther. 2000;30: 418–427. 10907898 10.2519/jospt.2000.30.7.418

[14] Green S. Cochrane handbook for systematic reviews of interventions version 5.1. 0 [updated March 2011]. The Cochrane Collaboration. 2011.

[15] Wells G, Shea B, O' Connell D, Peterson J, Welch V, M L. The Newcastle-Ottawa Scale (NOS) for assessing the quality of nonrandomized studies in meta-analyses 2013 [cited 2013 Sept 13]. Available: www.ohri.ca/programs/clinical_epidemiology/oxford.htm.

[16] Lewek M, Rudolph K, Axe M, Snyder-Mackler L. The effect of insufficient quadriceps strength on gait after anterior cruciate ligament reconstruction. Clin Biomech (Bristol, Avon). 2002;17: 56–63. 10.1016/s0268-0033(01)00097-3 11779647

[17] Reider B, Arcand MA, Diehl LH, Mroczek K, Abulencia A, Stroud CC, et al Proprioception of the knee before and after anterior cruciate ligament reconstruction. Arthroscopy. 2003;19: 2–12. 12522394 10.1053/jars.2003.50006

[18] Hewett TE, Myer GD, Ford KR, Heidt RS, Colosimo AJ, McLean SG, et al Biomechanical measures of neuromuscular control and valgus loading of the knee predict anterior cruciate ligament injury risk in female athletes A prospective study. Am J Sports Med. 2005;33: 492–501. 15722287 10.1177/0363546504269591

[19] Sell TC, Ferris CM, Abt JP, Tsai YS, Myers JB, Fu FH, et al Predictors of proximal tibia anterior shear force during a vertical stop‐jump. J Orthop Res. 2007;25: 1589–1597. 17626264 10.1002/jor.20459

[20] Benjuya N, Plotqin D, Melzer I. Isokinetic profile of patient with anterior cruciate ligament tear. Isokinet Exerc Sci. 2000;8: 229–232.

[21] Dvir Z, Eger G, Halperin N, Shklar A. Thigh muscle activity and anterior cruciate ligament insufficiency. Clin Biomech (Bristol, Avon). 1989;4: 87–91. 10.1016/0268-0033(89)90044-2 23915999

[22] Eitzen I, Moksnes H, Snyder-Mackler L, Risberg MA. A progressive 5-week exercise therapy program leads to significant improvement in knee function early after anterior cruciate ligament injury. J Orthop Sports Phys Ther. 2010;40: 705–721. 10.2519/jospt.2010.3345 20710097 PMC3158986

[23] Friden T, Zatterstrom R, Lindstrand A, Moritz U. Disability in anterior cruciate ligament insufficiency. An analysis of 19 untreated patients. Acta Orthop Scand. 1990;61: 131–135. 2360429 10.3109/17453679009006504

[24] Kannus P. Ratio of hamstring to quadriceps femoris muscles' strength in the anterior cruciate ligament insufficient knee. Relationship to long-term recovery. Phys Ther. 1988;68: 961–965. 3375319 10.1093/ptj/68.6.961

[25] Keays SL, Bullock-Saxton JE, Newcombe P, Keays AC. The relationship between knee strength and functional stability before and after anterior cruciate ligament reconstruction. J Orthop Res. 2003;21: 231–237. 12568953 10.1016/S0736-0266(02)00160-2

[26] Lee HM, Cheng CK, Liau JJ. Correlation between proprioception, muscle strength, knee laxity, and dynamic standing balance in patients with chronic anterior cruciate ligament deficiency. Knee. 2009;16: 387–391. 10.1016/j.knee.2009.01.006 19239988

[27] Lee JC, Kim JY, Park GD. Effect of 12 Weeks of Accelerated Rehabilitation Exercise on Muscle Function of Patients with ACL Reconstruction of the Knee Joint. J Phys Ther Sci. 2013;25: 1595–1599. 10.1589/jpts.25.1595 24409028 PMC3885847

[28] Lephart SM, Perrin DH, Fu FH, Gieck JH, McCue FC, Irrgang JJ. Relationship between Selected Physical Characteristics and Functional Capacity in the Anterior Cruciate Ligament-Insufficient Athlete. J Orthop Sports Phys Ther. 1992;16: 174–181. 18796757 10.2519/jospt.1992.16.4.174

[29] Segawa H, Omori G, Koga Y, Kameo T, Iida S, Tanaka M. Rotational muscle strength of the limb after anterior cruciate ligament reconstruction using semitendinosus and gracilis tendon. Arthroscopy. 2002;18: 177–182. 11830812 10.1053/jars.2002.29894

[30] Tsepis E, Vagenas G, Giakas G, Georgoulis A. Hamstring weakness as an indicator of poor knee function in ACL-deficient patients. Knee Surg Sports Traumatol Arthrosc. 2004;12: 22–29. 14586488 10.1007/s00167-003-0377-4

